# A proliferation-dependent bystander effect in primary porcine and human urothelial explants in response to targeted irradiation

**DOI:** 10.1038/sj.bjc.6600804

**Published:** 2003-03-04

**Authors:** O V Belyakov, M Folkard, C Mothersill, K M Prise, B D Michael

**Affiliations:** 1 Gray Cancer Institute, PO Box 100, Mount Vernon Hospital, Northwood, Middlesex HA6 2JR, UK; 2 Radiation and Environmental Science Centre, Dublin Institute of Technology, Kevin Street, Dublin 8, Ireland

**Keywords:** bystander effect, microbeam, urothelium, explant, proliferation

## Abstract

The aim of this study was to test whether radiation-induced bystander effects are involved in the response of multicellular systems to targeted irradiation. A primary explant technique was used that reconstructed the *in vivo* microarchitecture of normal urothelium with proliferating and differentiated cells present. Sections of human and porcine ureter were cultured as explants and irradiated on day 7 when the urothelial outgrowth formed a halo around the tissue fragment. The Gray Cancer Institute charge particle microbeam facility allowed the irradiation of individual cells within the explant outgrowth with a predetermined exact number of ^3^He^2+^ ions (which have very similar biological effectiveness to *α*-particles). A total of 10 individual cell nuclei were irradiated with 10 ^3^He^2+^ ions either on the periphery, where proliferating cells are located, or at the centre of the explant outgrowth, which consisted of terminally differentiated cells. Samples were fixed 3 days after irradiation, stained and scored. The fraction of apoptotic and micronucleated cells was measured and a significant bystander-induced damage was observed. Approximately 2000–6000 cells could be damaged by the irradiation of a few cells initially, suggesting a cascade mechanism of cell damage induction. However, the fraction of micronucleated and apoptotic cells did not exceed 1–2% of the total number of the cells within the explant outgrowth. It is concluded that the bystander-induced damage depends on the proliferation status of the cells and can be observed in an *in vitro* explant model.

Until recently, it has been commonly accepted that the biological consequences following radiation exposure are attributable to direct DNA damage. According to this paradigm, DNA damage occurs during or very shortly after irradiation of the nuclei in targeted cells, and the potential for biological consequences can be expressed within one or two cell generations (Little, 2000). Several lines of evidence have now emerged that challenge the idea that the biological effects result from targeted damage to DNA. These new effects have been termed ‘nontargeted’ and include radiation-induced bystander effects, genomic instability, low-dose hypersensitivity and adaptive responses (Ward, 1999). A common feature of ‘nontargeted’ effects is that they are significant responses at low doses of relevance to fractionated radiotherapy and protection level exposures.

The radiation-induced bystander effect is a phenomenon whereby cellular damage (sister chromatid exchanges (Nagasawa and Little, 1992; Lehnert and Goodwin, 1997), chromosome aberrations (Little *et al*, 1997; Lorimore *et al*, 1998), apoptosis (Mothersill and Seymour, 1997; Mothersill *et al*, 2000), micronucleation (Prise *et al*, 1998; Belyakov *et al*, 2001), transformation (Sigg *et al*, 1997), mutations (Zhou *et al*, 2000) and changes of gene expression (Hickman *et al*, 1994; Azzam *et al*, 1998)) are expressed in unirradiated neighbouring cells close to an irradiated cell or cells. The mechanisms underpinning the bystander effect are not yet known. However, there is evidence that the bystander effect may have at least two separate pathways for the transfer of damage from irradiated cells to unirradiated neighbours: through gap junctions or by cell-culture-mediated factors.

Several studies (Azzam *et al*, 1998,^2001^) have demonstrated that the bystander effect is dependent on gap junction intercellular communication (GJIC) in confluent cultures of primary human diploid fibroblasts exposed to low fluences of *α*-particles. These showed that p53 and p21 mediated pathways are activated (Azzam *et al*, 2000). Other studies reported that a p53-mediated signalling pathway could be activated in the bystander effect (Hickman *et al*, 1994), after low-dose *α*-particle irradiation of rat lung epithelial cells. Flow cytometric analysis of the fraction of cells with elevated levels of p53 protein detected an increased expression in a higher proportion of cells than were hit by an *α*-particle.

The other proposed mechanism of the bystander effect is mediation by secretion of factors into the culture medium (Mothersill and Seymour, 1997). A series of studies (Narayanan *et al*, 1997) suggests a mechanism in which the irradiated cells secrete cytokines or other factors that act to increase intracellular levels of reactive oxygen species in unirradiated cells (Iyer and Lehnert, 2000b). In particular, it was demonstrated that the culture medium harvested from the cells irradiated with low fluences of *α*-particles could induce an increase in sister chromatid exchanges when incubated with unirradiated test cells. For reactive oxygen species, a role for superoxide and hydrogen peroxide has been reported, although these may only be downstream consequences of bystander initiation. The elimination of the bystander effect by heat treatment of the harvested medium or by treatment of irradiated cells with protein synthesis inhibitors suggests that the secreted factors could be proteins (Lehnert and Goodwin, 1997).

Little is known regarding the role of bystander effects in multicellular systems. The radiosensitivity of HPV-G and HaCaT epithelial cell lines irradiated within microcolonies (>50 cells) was found to be lower than when they were irradiated as single cells (Cummins *et al*, 1999). A recent study (Bishayee *et al*, 1999) detected a pronounced bystander effect in a V79 three-dimensional (3D) tissue culture model labelled with ^3^H when the isotope was localised in the cell nucleus and distributed nonuniformly among the cells. Other studies (Jen *et al*, 1991) have found that the radiosensitivity of mouse kidney cells that were irradiated under *in vivo* conditions *in situ* or *in vitro* as fragments was higher than those irradiated *in vitro* as single cells. More recently, irradiated haemopoietic stem cells were observed to produce a bystander response *in vivo* when these were transplanted back into animals (Watson *et al*, 2000).

Our own studies (Prise *et al*, 1998; Belyakov *et al*, 2001) demonstrated that irradiation of a single human fibroblast with a single ^3^He^2+^ particle produced a significant bystander effect with a 2–3-fold increase in the micronucleated and apoptotic cells fraction in the surrounding unirradiated population. Further increases of dose to the irradiated cell did not increase the number of cells responding. The aim of this study was to test whether bystander responses are induced in a primary tissue model where individual cells had been targeted with radiation. For this, we utilised a ureter explant system developed from either human or porcine samples.

## MATERIALS AND METHODS

### Ureter samples

Human ureter samples were obtained for Dr C Mothersill, from consenting patients undergoing reconstructive surgery for benign conditions at local hospitals. The studies had ethical approval from the hospitals' ethics committees and from the Dublin Institute of Technology (DIT). Samples were placed in sterile physiological saline immediately on removal from the patient and shipped on ice to the laboratory. Explants were normally established within 24 h.

Dr Mohi Rezvani and Neil Hubbard (Churchill Hospital, University of Oxford) generously provided porcine ureter samples. They were obtained from 10 to 72-week-old farm pigs during post-mortem examination. These animals were maintained in compliance with the Animal (Scientific Procedures) Act 1986 (Workman *et al*, 1998). Samples were placed in sterile ‘transport medium’ after removal and shipped on ice. Transport medium (Southgate *et al*, 1995) was based on RPMI 1640 with L-glutamine (Sigma, Poole, UK) containing 20 mM HEPES, 10% (v v^−1^) foetal calf serum (Sigma, Poole, UK) and 20 IU ml^−1^ of aprotinin (Sigma, Poole, UK) with additions of penicillin (100 IU ml^−1^) and streptomycin (100 *μ*g ml^−1^), (50 *μ*g ml^−1^) fungisone (Gibco, Paisley, UK) and nystatin (Sigma, Poole, UK). Explants were normally established within 24 h.

### Primary explant technique

An explant approach (Mothersill, 1998) was used for studying bystander effects under *in vivo*-like conditions where proliferating and differentiated cells were present. Ureter samples were trimmed from fat and connective tissue, opened with fine scissors longitudinally and cut into segments of approximately 2–3 mm^2^ for explantation. Samples were treated with a 0.25% (w v^−1^) trypsin solution (Gibco, Paisley, UK) containing 10 mg ml^−1^ collagenase IV (Sigma, Poole, UK) in Hank's balanced solution (Sigma, Poole, UK) and incubated for 30 min at 37°C. Ureter fragments were then plated into specially designed dishes (Folkard *et al*, 1997b) consisting of a 34 mm diameter base composed of a 1.5 *μ*M thick mylar membrane (Goodfellow, Cambridge, UK) for microbeam experiments or in T25 tissue culture flasks (Primaria, Falcon, Marathon Lab Supplies, London, UK) for cell growth measurements and BUdR cell proliferation measurements. The dishes contained 2 ml of serum rich ‘start-up’ medium for initial outgrowth formation. This was based on RPMI 1640 with L-glutamine (Sigma, Poole, UK), containing 13% (v v^−1^) foetal calf serum and 7% (v v^−1^) horse serum (Sigma, Poole, UK) with additions of 100 mIU insulin (Sigma, Poole, UK), 1 mg ml^−1^ hydrocortisone (Sigma, Poole, UK), 30 ng ml^−1^ human recombinant EGF (Sigma, Poole, UK), penicillin (100 IU ml^−1^) and streptomycin (100 *μ*g ml^−1^), (50 *μ*g ml^−1^) fungisone (Gibco, Paisley, UK) and nystatin (Sigma, Poole, UK).

**Figure 1:**
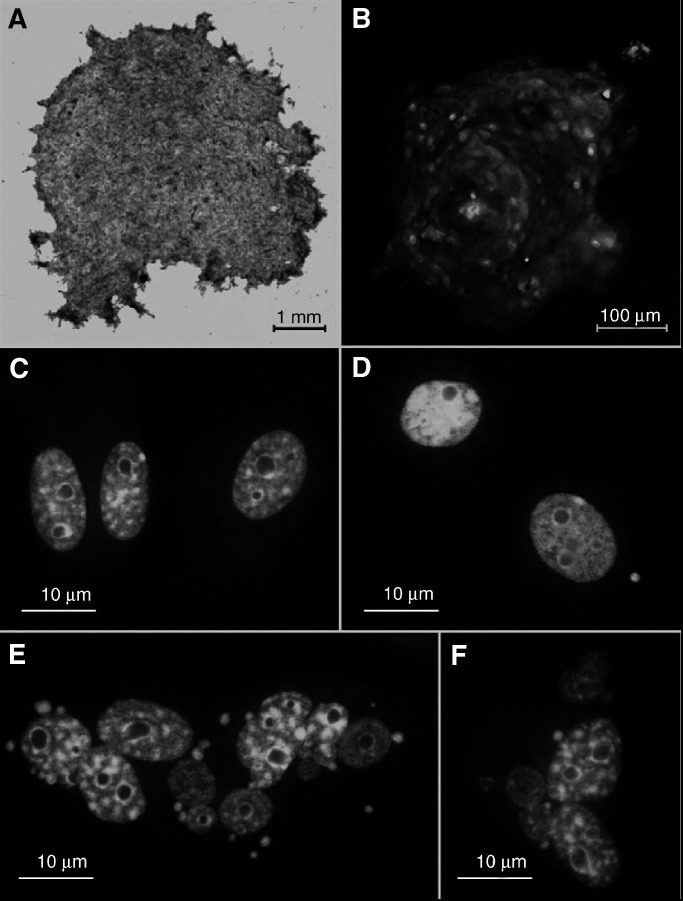
(**A**) Phase contrast image of a 7-day-old human urothelium explant outgrowth. (**B**) Human ureter outgrowth stained with pan anticytokeratin antibodies, FITC/PI staining at day 3. Images of normal porcine urothelial cells within the explant outgrowth (**C**), micronucleated cells (**D**), and apoptotic cell (**E**, **F**), all stained with acridine orange.

After 2–3 days of incubation (37°C in an atmosphere of 95% air and 5% CO_2_) the medium was replaced with serum-free Keratinocyte Growth Medium (Clonetics, UK) or Keratinocyte-SFM (Gibco, Paisley, UK) and incubated for a further 4–5 days. A typical 7-day-old, human ureter urothelium outgrowth was a few millimetres in diameter (Figure 1A

) and consisted mainly of a monolayer although with a few dense regions. The ‘two media’ technique was developed to avoid contamination of the outgrowth with fibroblasts and to promote differentiation of the urothelial cells (Mothersill, 1998). Staining with pan anticytokeratin antibodies (Sigma, Poole, UK) was used in selected cases to check the explant outgrowth for fibroblast contamination following the method of Hutton *et al* (1993). Cytokeratin immunostaining is a marker for epithelial cell lines. Pan cytokeratin FITC conjugated antibodies (Monoclonal clone no. C 11, mouse, IgG1, Sigma, Poole, UK) were used to visualise the marker (Figure 1B). At the time of irradiation on day 7, fibroblasts were completely eliminated from the outgrowth.

### Microbeam irradiation

The Gray Cancer Institute charged particle microbeam (Folkard *et al*, 1997a,1997b) allowed the irradiation of single cells with a precise number of particles. All irradiations were performed with ^3^He^2+^ ions (3.5 MeV; LET 100 keV *μ*m^−1^), which have almost identical track structure to that of *α*-particles. The explant outgrowth was irradiated through the base of the microbeam dish.

**Figure 4:**
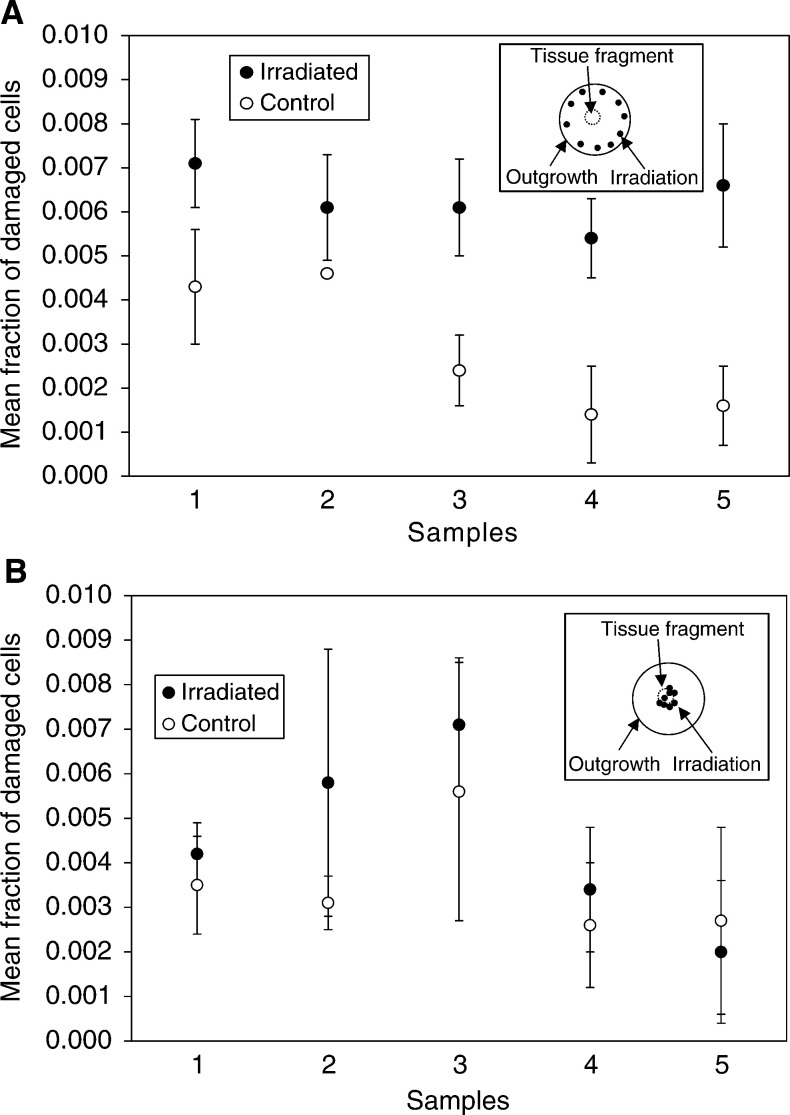
(**A**) Fraction of damaged cells after microbeam irradiation at the periphery of porcine urothelial explant outgrowth, 10 cells were irradiated at the edge of each explant (10 ^3^He^2+^ particles per cell). (**B**) Fraction of damaged cells after microbeam irradiation at the centre of porcine urothelial explant outgrowth, 10 cells have been irradiated at the centre of each explant (10 ^3^He^2+^ particles per cell). Samples were fixed, stained and scored on day 3 after irradiation. Error bars represent standard deviation of the means.

Cell nuclei were visualised by staining with 1 *μ*M Hoechst 33258 (Sigma, Poole, UK) in KGM medium for 1 h before irradiation. During the irradiation, the ureter explant outgrowth was incubated with 20 *μ*M HEPES KGM-based medium at room temperature. A total of 10 individual cell nuclei were irradiated each with 10 ^3^He^2+^ particles in a 7-day-old explant outgrowth. Cell nuclei to be irradiated were randomly selected at the periphery (Figure 4A

, inset) or at the centre of the outgrowth (Figure 4B, inset) and then positioned over the collimator using the microbeam stage. The particles were delivered to a single location at the centre of each nucleus with high precision (>99% within 2 *μ*m) and the number of delivered particles was counted with a particle detector (Folkard *et al*, 1997a,1997b). The irradiation procedure typically took about 15 min after which samples were incubated in fresh KGM medium at 37°C in 95% air and 5% CO_2_ for up to 3 days prior to scoring. Control dishes were treated in exactly the same way but not irradiated. Typically, an experimental set consisted of four irradiated and two control explants originating from the same sample.

### Scoring of micronucleated and apoptotic cells

On day 3 after irradiation the samples were washed in phosphate-buffered saline (PBS), fixed in 100% methanol and stained for 20 min with 0.5% (w v^−1^) acridine orange (Sigma, Poole, UK), destained in PBS for 1 h, air-dried and scored using a fluorescent microscope. Typical normal urothelial cells are shown in (Figure 1C). Samples were scored for the presence of micronucleated (Figure 1D) and apoptotic (Figure 1E, F) cells measured as total cell damage (Abend *et al*, 1995, 2000). Micronuclei appeared as green-coloured round bodies separated from the main nucleus as previously described (Belyakov *et al*, 1999). Apoptotic cells were classified on the basis of morphological criteria (Kerr *et al*, 1972). The number of cells with micronuclei and apoptotic cells were determined for each dish. During the scoring only the micronucleated and apoptotic cells were registered using a Zeiss-Axioskope fluorescent microscope and a cooled CCD camera system (Photonic Science, UK). The total number of cells within an explant outgrowth was estimated by measuring the explant size using a specially constructed imaging system (Vojnovic, 1996). The total number of cells within the explant outgrowth was calculated using random measurements of cell density per 100 *μ*m^2^ for each individual explant outgrowth. Taken together, this allowed estimates of the growth kinetics of the explant outgrowth to be made. The spatial distribution of cell damage was assessed on selected samples. It was obtained by scanning a straight line across an explant in 0.1 mm steps and counting the number of damaged and the total number of cells in each field of view (typically 80–120 cells per field of view). Fractions of micronucleated and apoptotic cells were calculated per field of view from one side of the explant to the other.

Cell proliferation was measured by adding 10 *μ*M BUdR (Sigma, Poole, UK) in culture medium for 3 days, to assess the total number of cells undergoing division after irradiation and before fixing 3 days later. Samples were stained with anti-BUdR (mouse IgG1) antibodies (Sigma, Poole, UK) and visualised with FITC mouse IgG (whole molecule) conjugated antibodies (Sigma, Poole, UK). A few explants were examined using antibodies to Uroplakin III, a specific marker of terminal urothelial differentiation on day 7. Explants were fixed and stained with Uroplakin III antibodies (Reseach Diagnostics, Inc., USA) and visualised with FITC conjugated antibodies (Sigma, Poole, UK). Scoring of the cells was performed by scanning a straight line across the explant in 0.1 mm steps under low magnification. The fractions of BUdR or Uroplakin III-positive cells were calculated per field of view.

### Statistical analysis

Cell damage data represent the mean and standard deviation for between two and four individual explants for each sample. In the case of cell kinetics, 10 individual explants were measured from each sample, with the mean and the standard error calculated. Individual explants were all number-coded and were scored blind. Significance tests were made using the Student's *t*-test.

## RESULTS

In this study, we have used both human and porcine urothelial explant outgrowths and compared growth dynamics, patterns of proliferation and differentiation. Cell growth assays were performed in parallel to microbeam irradiation experiments. Micro-beam irradiations were performed on day 7 when the explant outgrowth was in an exponential growth phase and consisted predominantly of quiescent cells (results not shown). The explant outgrowth doubled in size within 2–3 days. The doubling time for primary urothelial cell under stimulated *in vitro* cultivation conditions is about 56±5.6 h (Petzoldt *et al*, 1994). However, in the explant outgrowth, only a small fraction (about 10%) of the cells was actively proliferating.

**Figure 2:**
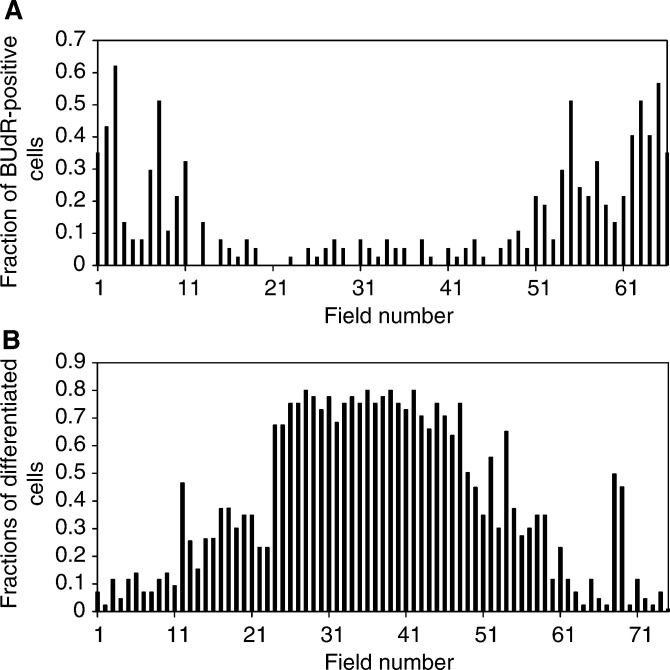
(**A**) BUdR cell proliferation assay with porcine urothelium explant outgrowth. The graph represents the spatial distribution of BudR-positive cells within a cross-section of a 10-day-old porcine urothelium explant outgrowth. Cells were scored across the explant in 0.1 mm steps. (**B**) Fraction of differentiated cells measured with Uroplakin III immuno-staining in porcine urothelial explant outgrowths within a cross-section of a control, 10-day-old porcine urothelium explant outgrowth.

A BUdR cell proliferation assay demonstrated that the proliferating cells were concentrated on the periphery of the explant outgrowth (Figure 2B

). This pattern essentially reconstructs in two dimensions the normal 3D microarchitecture of urothelium *in vivo* (Reznikoff *et al*, 1983). Urothelial differentiation was measured in nonirradiated samples by immunostaining with antibodies against Uroplakin III. This is a specific marker for terminal urothelial differentiation and staining was much more specific than in the case of earlier pilot experiments that used an array of WGA, DBA and PNA lectins (Sigma, Poole, UK, according to the methods developed by Fujiyama *et al* (1995) data not shown). A typical example of the nonirradiated pattern of differentiation within the urothelial explant outgrowth is represented in Figure 2C. It can be clearly seen that differentiated cells tend to concentrate at the centre of the outgrowth. Normally, 50–70% of cells within a mature urothelial explant outgrowth would be differentiated under the conditions used here. No significant differences in the growth kinetics and patterns of proliferation and differentiation were observed between the human or porcine samples.

**Figure 3:**
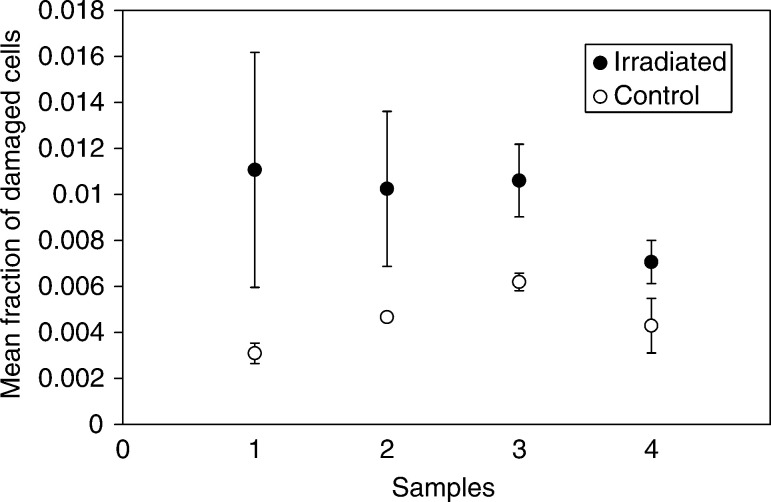
Fraction of damaged cells after microbeam irradiation at the periphery of a human urothelial explant outgrowth. A total of 10 cells were irradiated at the edge of each explant (10 ^3^He^2+^ particles per cell). Samples were fixed, stained and scored on day 3 after irradiation. Error bars represent standard deviation of the means. Each sample was from a separate donor ureter.

The results of experiments with localised irradiation of 10 cells spaced at the periphery of human urothelium explant outgrowth, each with 10 ^3^He^2+^ particles is shown in Figure 3

. The fraction of damaged (apoptotic and micronucleated) cells was considerably higher in irradiated explants in comparison with controls. Typically, 10% of the damaged cells were apoptotic, according to the morphological criteria. The fraction of damaged cells was obtained by dividing of the number of apoptotic and micronucleated cells within the entire explant by the total number of cells scored. There was no significant difference, in the fraction of micronucleated and apoptotic cells present, between explants, which had not been exposed to radiation, and dishes containing explants where only the medium was irradiated (data not shown). A significant variation in the background levels of micronucleated and apoptotic cells for different samples was observed, which is typical for primary samples because of genetic and age-dependent variations between individuals. Even although only 10 cells were irradiated at the actively proliferating edge of the human explant outgrowth, up to a several thousand additionally micronucleated and apoptotic cells (1700–5700) were observed 3 days after irradiation (Table 1

**Table 1:** Results of experiments of irradiation with 10 ^3^He^2+^ particles of 10 individual cell nuclei each distributed on the periphery of a 7-day-old human ureter explant outgrowth

	**Control**			**Irradiated**			
**Sample**	**Mean number of damaged cells**	**Mean calculated number of cells within explant outgrowth (*m*_C_)**	**Mean fraction of damaged cells ± s.d. (*f*_C_)**	**Mean number of damaged cells**	**Mean calculated number of cells within explant outgrowth (*m*_I_)**	**Mean fraction of damaged cells ± s.d. (*f*_I_)**	**Mean calculated number of additionally damaged cells^a^**
1	2433	7.8 × 10^5^	0.0031±0.0004	7074	6.4 × 10^5^	0.0111±0.0051	5689
2	3279	7.0 × 10^5^	0.0047^b^	6267	6.1 × 10^5^	0.0102±0.0032	3608
3	3075	5.0 × 10^5^	0.0062±0.0004	5217	4.9 × 10^5^	0.0106±0.0022	2174
4	2604	6.1 × 10^5^	0.0043±0.0012	4215	5.9 × 10^5^	0.0071±0.0015	1679

a Mean number of additionally damaged cells is equal to (*f*_I_−*f*_C_) × (*m*_C_+*m*_I_)/2.

b Single explant only.

). The mean number of additional damaged cells was calculated by subtraction of the mean background fraction of micronucleated and apoptotic cells from the mean fraction for irradiated explants within one sample (derived from the same individual) and multiplied by the mean calculated number of cells within the explant outgrowth for this sample.

In the rest of this study, porcine explants were used, with individual urothelial cells irradiated within the 7-day-old explants located either on the periphery, where proliferating cells are located or at the centre of the explant outgrowth, which consisted of terminally differentiated cells. The results of experiments where 10 cells were irradiated at the periphery of porcine urothelium explant outgrowth with 10 ^3^He^2+^ particles are shown in Figure 4A. Five samples from five different pigs were examined. The fractions of damaged cells (apoptotic and micronucleated) were scored on day 3 after irradiation. Irradiated explants demonstrated considerably higher numbers of damaged cells in comparison with the controls (Table 2

**Table 2:** Results of experiments of irradiation with 10 ^3^He^2+^ particles of 10 individual cell nuclei each distributed on the periphery of a 7-day-old porcine ureter explant outgrowth

	**Control**			**Irradiated**			
**Sample**	**Mean number of damaged cells**	**Mean calculated number of cells within explant outgrowth (*m*_C_)**	**Mean fraction of damaged cells ± s.d. (*f*_C_)**	**Mean number of damaged cells**	**Mean calculated number of cells within explant outgrowth (*m*_I_)**	**Mean fraction of damaged cells ± s.d (*f*_I_)**	**Mean calculated number of the additionally damaged cells^a^**
1	2663	6.2 × 10^5^	0.0043±0.0013	4215	5.9 × 10^5^	0.0071±0.001	1698
2	2093	4.5 × 10^5^	0.0046^b^	2617	4.3 × 10^5^	0.0061±0.0012	663
3	2039	8.5 × 10^5^	0.0024±0.0008	4874	8.0 × 10^5^	0.0061±0.0011	3050
4	918	6.6 × 10^5^	0.0014±0.0011	3568	6.6 × 10^5^	0.0054±0.0009	2633
5	1257	7.9 × 10^5^	0.0016±0.0009	5236	7.9 × 10^5^	0.0066±0.0014	3948

a Mean number of additionally damaged cells is equal to (*f*_I_−*f*_C_) × (*m*_C_+*m*_I_)/2.

b Single explant.

). Interestingly, the overall response to micro-beam irradiation of porcine ureter explant outgrowth was not considerably different from those obtained with the human samples (both irradiated at the periphery). Again, a significant interindividual variation in the response was observed.

In contrast, we did not get a statistically different level of micronucleated and apoptotic cells after irradiation of 10 cells at the centre of the porcine urothelial explant outgrowth, where mainly terminally differentiated cells are present (Figure 4B). Five samples from five individual pigs (different from those of the previous set) were examined. Larger standard deviations in comparison with the set of data with irradiation of the explant periphery suggest a less uniform response, which may be due to the presence of some proliferating cells within the centre of the outgrowth (Figure 2B). Overall, our finding demonstrates that 10 cells irradiated at the actively proliferating edge of a porcine explant outgrowth produce up to 600–4000 additionally micronucleated and apoptotic cells 3 days after irradiation (Table 2).

**Figure 5:**
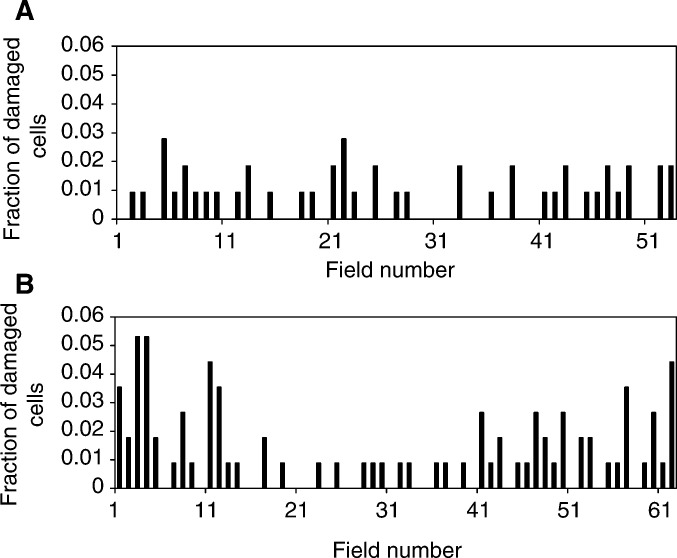
(**A**) Spatial distribution of the damaged cells in control porcine ureter explant outgrowth is shown. (**B**) Spatial distribution of the damaged cells after microbeam irradiation of 10 individual cells, each with 10 ^3^He^2+^ particles selected at the periphery of the porcine ureter explant outgrowth. Fraction of damaged per field of view, across the explant in 0.1 mm steps is plotted.

The spatial distribution of damaged cells within the explants was assessed on selected porcine and human samples. Fractions of damaged cells were calculated per field of view, scanning a straight line across an explant. It was demonstrated that background damaged cells are distributed uniformly throughout the explant outgrowth (Figure 5A

). Microbeam irradiation of 10 cells on an actively proliferating edge resulted in additional damaged cells that were concentrated mainly at the periphery of the explant outgrowth (Figure 5B). Spatial distribution of cellular damage after irradiation of 10 cells at the centre of an urothelial explant outgrowth was similar to the background cell damage distribution in the control sample.

**Figure 6:**
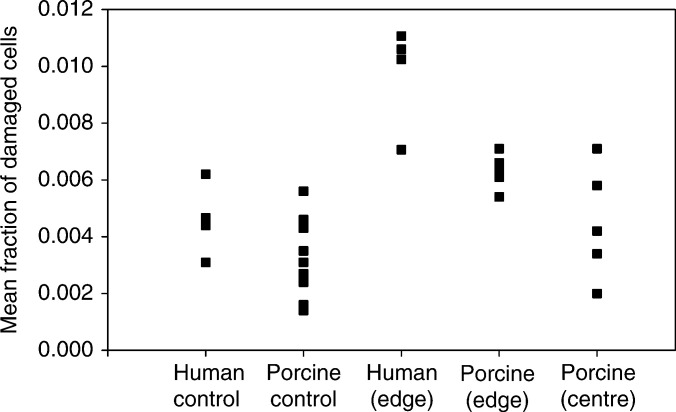
Interindividual variations of the bystander response in human and porcine samples. All squares represent the means of the total cellular damage fractions for every individual sample. Human and porcine controls are compared with human or porcine samples irradiated at the periphery (edge) and porcine samples irradiated at the centre.

There was considerable interindividual variations of bystander response in human and porcine samples (Figure 6

). Generally, the human samples demonstrated a higher value of bystander response in comparison with porcine samples (statistically significant for *P*<0.05). However, control mean values for human samples are not statistically different from the porcine ones (*P*<0.05). Porcine samples irradiated at the centre demonstrated considerably higher variation in response than those irradiated at the periphery.

## DISCUSSION

Most of the studies on radiation-induced bystander effects have been performed with *in vitro* cell culture models (Iyer and Lehnert, 2000a). The aim of this approach was to test the role of bystander response in an *in vivo*-like multicellular system using a microbeam technique for targeting individual cells. An urothelium explant outgrowth system was developed to study bystander effects in a model where dividing and differentiated cells were present. The ureter provides a useful model system for tissue studies. The epithelial layer is highly organised with clear functional delineation between stem, dividing and functionally differentiated cells (Southgate *et al*, 1995). In the explant model used here, we can reconstruct, in two dimensions, the organisational structure present in three dimensions in the original tissue. The growing explant has an outer region of dividing cells with terminal differentiation occurring toward the centre.

In this model, we have demonstrated evidence for a radiation-induced bystander effect, in both primary human (Figure 3 and Table 1) and porcine (Figure 4A and Table 2) urothelial outgrowth explant systems. The bystander response was found in the explant outgrowth after irradiation of 10 cells, each with 10 individual ^3^He^2+^ ions, located on the periphery, where actively proliferating cells are found. In terms of absolute numbers, an additional 700–5700 cells could be affected by targeting of only 10 cells, suggesting a cascade mechanism for damage induction. Overall, the bystander effect we report here is small, when taken as a fraction of the total cells within the explant outgrowth, accounting for a two-fold increase in the background frequency of damage. In general, a higher level of bystander response was observed in the human relative to the porcine samples, although there were no significant differences in the morphology and growth characteristics between the two species.

The bystander effect in an *in vivo*-like urothelial explant system appears to be more substantial than in a purely *in vitro* primary fibroblast system after targeted low-dose microbeam irradiation as we reported recently (Prise *et al*, 1998; Belyakov *et al*, 2001). Those studies were performed with cells at low densities where cell-to-cell contact was minimal and therefore a media-related factor was involved. However, other studies in confluent monolayers of fibroblasts showed that GJIC also played a role in propagation of the bystander effect (Azzam *et al*, 1998,^2001^). Urothelium has highly developed connexin-mediated intercellular communication (Grossman *et al*, 1994; Lyng *et al*, 1996) and this could explain the more pronounced bystander effect observed in the urothelial outgrowth in comparison with fibroblast cultures.

The results of our microbeam experiments with irradiation of the explant outgrowth strongly suggest that the proliferative or differentiation state is important for manifestation of the bystander effect. Other evidence towards this conclusion is that micronucleated and apoptotic cells were distributed nonrandomly across the entire area of the explant outgrowth after irradiation of the explant periphery. Damaged cells appeared towards the edges of the outgrowth (Figure 5B). Control samples (Figure 5A) demonstrated a more uniform distribution of the background micronucleated and apoptotic cells, and irradiation of cells at the centre of the explant does not change the spatial distribution in comparison with the control.

Some studies have also suggested that radiation-induced bystander effects may be related to radiation-induced genomic instability (Lorimore *et al*, 1998; Watson *et al*, 2000). One potential conclusion from the ureter studies reported here is that the micronucleated and apoptotic cells observed in the explant outgrowth are simply due to the induction of genomic instability in targeted stem or dividing cells. A dose of 10 helium ions to a urothelial cell within the explant outgrowth is equivalent to ∼1 Gy with possibly a >50% probability of killing the targeted cell. If out of 10 cells irradiated, five cells survived and exhibited instability in the surviving progeny leading to chromosomal damage, these cells would have to divide through ∼8–10 generations to produce the observed numbers of damaged cells, assuming a high probability of a micronucleated or apoptotic cells being produced. Also, under these conditions it would have been predicted that the micronucleated and apoptotic cells would be located physically close to the originally targeted cell. The typical doubling time of the explants used here is around 2–3 days, and only around 10% of the explant is dividing (from BUdR labelling). Given this and the fact that no evidence of clustering of micronucleated and apoptotic cells is observed, it is unlikely that the majority of the effect we have observed is due solely to induction of genomic instability.

There are interindividual variations in the measured bystander response (Figure 6), which might be explained by different genetic and physiological backgrounds of the sample donors although we do not have specific information on markers, which may be relevant within the limited number of samples presented here. This raises an important question of individual susceptibility to bystander responses. It has been demonstrated that gap-junction-mediated communication in human bladder explant outgrowth depends on a smoking status of the tissue donors (Lyng *et al*, 1996). Also, other studies have reported a relationship between the level of bystander signal produced and gender and malignancy status (Mothersill *et al*, 2001). Further studies in defined populations using the model described here would clearly be useful to clarify the variations in response we have observed.

Our findings contribute to continuing debate regarding the relevance of *in vitro* cells culture systems to the multicellular tissue system. The role of intercellular communication (including bystander effects) under *in vivo* conditions might be highly individual and tissue specific. *In vitro* cell systems are unlikely to exactly mimic the *in vivo* system response in terms of carcinogenesis. To date, the only other data, where cells within multicellular systems have been targeted with radiation demonstrating bystander responses, have utilised models containing one cell type (Bishayee *et al*, 1999, 2000). Overall, understanding the role of bystander responses may be important, not just for determining the role of cell–cell communication in radiation responses, but may offer novel approaches to improving therapeutic strategies involving targeted radiotherapy regimens. For example, switching on damage-inducing bystander responses in tumour cells may improve the efficacy of targeted radiation approaches or combined gene therapy. Alternatively, it may be possible to protect normal tissues from responding by switching off bystander interactions.

In summary, we have demonstrated evidence of a radiation-induced bystander effect within human and porcine urothelium explant outgrowths where dividing and differentiated cells were present. The bystander response was observed when the actively proliferating region within an explant outgrowth was targeted and the distribution of additional micronucleated and apoptotic cells was nonuniform. This evidence strongly suggests that the expression of bystander damage and proliferation/differentiation state of the cells involved is linked. Further studies will test the underlying mechanisms that lead to signal transduction under these conditions.
